# Changes in Invasive Pneumococcal Disease in the Paediatric Population in the Second COVID-19 Pandemic Year

**DOI:** 10.3390/vaccines11101548

**Published:** 2023-09-29

**Authors:** Conchita Izquierdo, Pilar Ciruela, Núria Soldevila, Juan-Jose Garcia-Garcia, Sebastia Gonzalez-Peris, Alvaro Díaz-Conradi, Belen Viñado, Mariona F de Sevilla, Fernando Moraga-Llop, Carmen Muñoz-Almagro, Angela Domínguez, Barcino Working Group

**Affiliations:** 1 Agència de Salut Publica de Catalunya, 08005 Barcelona, Spain; pilar.ciruela@gencat.cat; 2 CIBER de Epidemiología y Salud Pública, 28029 Madrid, Spain; nsoldevila@ub.edu (N.S.); juanjose.garciag@sjd.es (J.-J.G.-G.); mariona.fernandez@sjd.es (M.F.d.S.); cma@sjdhospitalbarcelona.org (C.M.-A.); angela.dominguez@ub.edu (A.D.); 3 Departament de Salut Pública, Universitat de Barcelona, 08036 Barcelona, Spain; 4 Departament de Cirurgia i Especialitats Médico-Quirúrgiques, Universitat de Barcelona, 08907 Barcelona, Spain; 5 Hospital Sant Joan de Déu Barcelona, 08950 Esplugues de Llobregat, Spain; 6 Institut de Recerca Sant Joan de Déu, 08950 Esplugues de Llobregat, Spain; 7 Hospital Vall d’Hebron, 08035 Barcelona, Spain; sgonzalezperis@gmail.com (S.G.-P.); belen.vinado@vallhebron.cat (B.V.); fernandomoragallop@gmail.com (F.M.-L.); 8 Hospital HM Nens, HM Hospitales, 08009 Barcelona, Spain; adiazconradi@hmhospitales.com; 9 Departament de Medicina, Universitat Internacional de Catalunya, Sant Cugat del Vallés, 08195 Barcelona, Spain

**Keywords:** impact of COVID-19, invasive pneumococcal disease, pneumococcal 13-valent conjugate vaccine, non-pharmaceutical measures

## Abstract

Incidence of invasive pneumococcal disease (IPD) decreased worldwide in 2020, coinciding with the implementation of measures to reduce COVID-19 transmission. We evaluated the impact of the COVID-19 pandemic on healthcare demand and IPD in children in 2021 compared to the pre-pandemic period (2018–2019) and the early pandemic period (2020) in a study carried out during 2018–2021 in Catalonia. Incidence rates were compared by calculating the incidence rate ratio (IRR), and expressing percentage changes in IRR as (1-IRR)x100. Compared to 2018–2019, emergency room (ER) visits declined by 21% in 2021 (*p* < 0.001), mainly in the first quarter (−39%), and compared to 2020, ER visits increased by 22% in 2021 (*p* < 0.001), except in the first quarter. IPD incidence overall was 11.0 in 2018–2019 and 4.6 in 2021 (−58%, *p* < 0.001); the reduction in incidence was similar in the 0–4 age group and was higher in the first quarters. Compared to 2020, in 2021, IPD incidence decreased during the first quarter (−86%, *p* < 0.001), but increased from 0.0 to 1.2 in the second quarter (*p* = 0.02) and from 0.6 to 2.1 (*p=0.03*) in the fourth quarter. The decreased IPD incidence observed in 2021 compared to 2018–2019 (most especially in the first quarter) was greater than the decrease in healthcare demand and PCR test requests. Compared to 2020, IPD incidence decreased in the first quarter when a second state of alarm was in force. In 2021, compared to 2018–2019, there was a greater reduction in PCV13 serotypes than in non-PCV13 serotypes.

## 1. Introduction

The incidence of invasive pneumococcal disease (IPD), similarly to other infectious diseases, decreased worldwide in 2020, coinciding with the implementation of non-pharmacological preventive measures (masks, hand washing, physical distancing) aimed at reducing transmission of the SARS-CoV-2 virus [1,2]. 

In Catalonia, IPD incidence in children aged <5 years was 35.3/100,000 inhabitants in 2019, with 68.9% of cases caused by serotypes not covered by the 13-valent pneumococcal conjugate vaccine (PCV13) [3]. In 2020, compared to 2018–2019, IPD cases in children aged <18 years fell by 65% [4], affecting both PCV13 and non-PCV13 serotypes; the fall was more marked in the second quarter of 2020, coinciding with a lockdown period. 

During 2021, in parallel with the relaxation of non-pharmacological preventive measures, IPD incidence began to increase again, reaching pre-pandemic levels in the second and third quarters of 2021 [5,6,7].

It is not known whether the differences observed in IPD incidence in 2021 compared to the first pandemic year and the pre-pandemic period may have been affected by changes in healthcare demand or in disease diagnosis. Some authors [8] have demonstrated that the strict COVID-19 lockdown of 2020 in Spain had a significant impact on children’s nasopharyngeal microbiota, specifically on the streptococcus genus, but also indicated that the role played by those changes in the colonization and development of *Streptococcus pneumoniae*-related disease needs to be investigated. 

The aim of this study was to evaluate changes caused by the COVID-19 pandemic in healthcare demand, requests for diagnostic tests for pneumococcal disease, and IPD incidence in the population aged <18 years, comparing 2021, the early pandemic period (2020), and the pre-pandemic period (2018–2019).

## 2. Material and Methods

A prospective–retrospective study was carried out from 2018 to 2021 in 3 hospitals in Barcelona that provide care for 32% of the hospitalized paediatric population in Catalonia: Hospital Sant Joan de Déu (HSJD), Hospital Infantil Vall d’Hebron (HIVH), and Hospital Municipal de Nens (HMN).

A confirmed IPD case was defined as patient symptoms consistent with an infectious disease and *S. pneumoniae* isolation or polymerase chain reaction (PCR)-detection of *S. pneumoniae* DNA from a normally sterile site. 

Data were collected on the following variables: number of emergency room (ER) visits, number of PCR diagnostic tests requested for pneumococcus from a normally sterile site, confirmed cases of IPD, and distribution by serotype according to PCV13 vaccine coverage or non-coverage. The diagnostic technique was recorded as PCR and/or culture. Data on the number of PCR diagnostic tests requested for pneumococcus from a normally sterile site were collected at the HSJD and at the HIVH. Incidence per 100,000 person-years (PY) was calculated based on the annual catchment population of the 3 hospitals. The periods 2021, 2020, and 2018–2019 were compared by calculating the incidence rate ratio (IRR) and 95% confidence interval (CI) values, overall, by age group (0–4 years and 5–17 years), and by quarter. The IRR was calculated as the quotient between the incidence rate in different periods, and percentage changes in the IRR were expressed using the following formula: (1-IRR)x100. Differences were considered significant when the *p* value was less than 0.05.

Analyses were performed using R version 3.5.0 statistical software. 

## 3. Results

Paediatric ER visits numbered 225,031 in 2018, 229,256 in 2019, 148,637 in 2020, and 178,243 in 2021. Compared to the mean for 2018–2019, ER visit incidence in 2021 fell by 21% (*p* < 0.001); a very marked fall in the 5–17 age group (−38%; *p* < 0.001) was offset by a slight increase in the 0–4 age group (+4%; *p* < 0.001) (Table 1). Reduction overall was maintained in all quarters (*p* < 0.001) (Table 2). Compared to 2020, overall ER visits in 2021 increased by 22% (*p* < 0.001), with a very large increase in the 0–4 age group (+60%; *p* < 0.001) offset by a decrease in the 5–17 age group (−7%; *p* < 0.001) (Table 1). By quarters, the 2021 increase was greatest in the second (+104%; *p* < 0.001) and fourth (+65%; *p* < 0.001) quarters, whereas a significant decrease occurred in the first quarter (−30%; *p* < 0.001) (Table 2).

Requested PCR tests for pneumococcus from a normally sterile site showed no change in 2021 compared to 2018–2019 overall and by age group (Table 1). By quarter, there were significant decreases in the first and fourth quarters (−47% and −25%, respectively; *p* < 0.01) and increases in the second and third quarters (+42% each; *p* < 0.01). In 2021, compared to 2020, requested PCR tests increased by 23% (*p* < 0.001), but more markedly in the 0–4 age group (+38%; *p* < 0.001) (Table 1); by quarter, increases were observed in the second and third quarters (+88% and +86%, respectively; *p* < 0.001), and a decrease was observed in the first quarter (−46%; *p* < 0.001) (Table 2).

**Figure 1:**
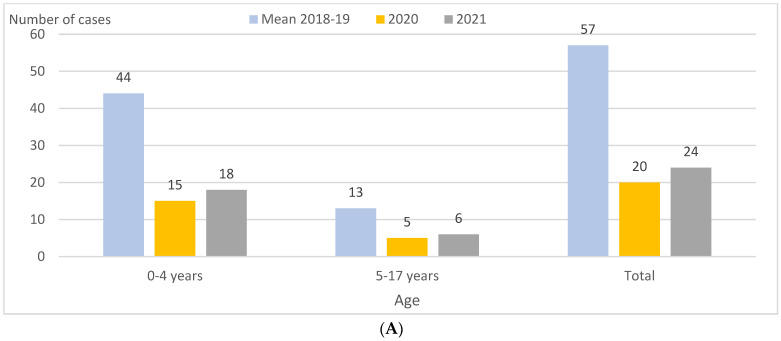

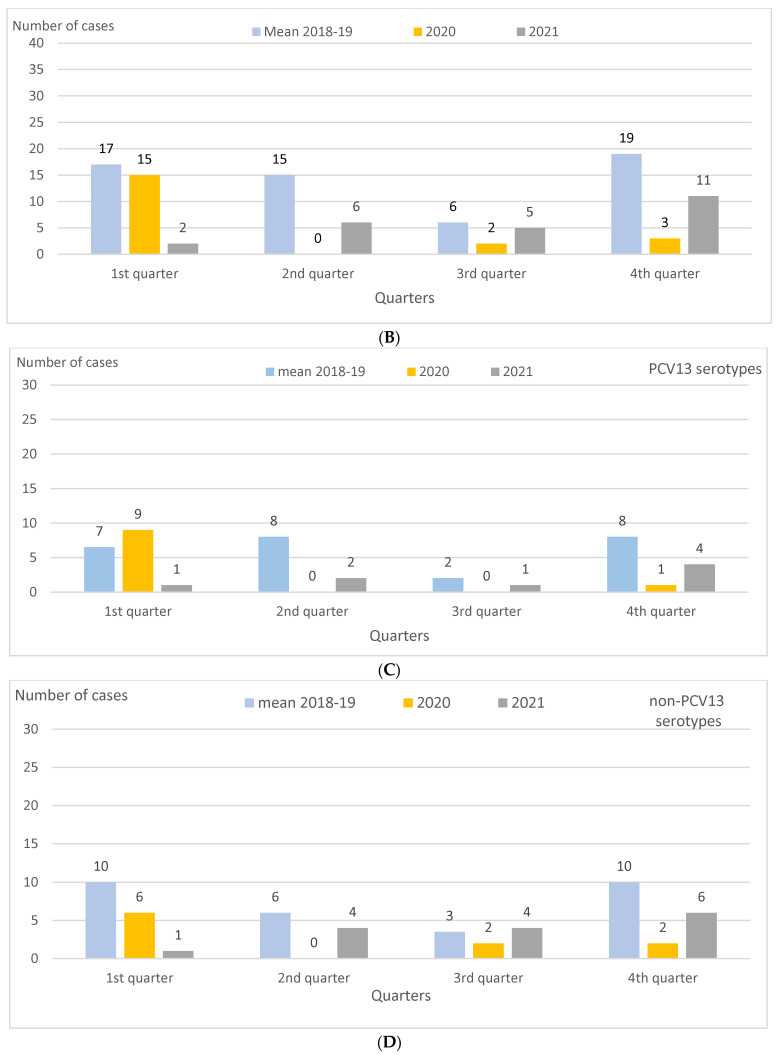
Children aged 0–17 years in Catalonia, Spain. IPD cases by age group, quarter, and PCV13 and non-PCV13 serotypes in 2018–2019 (mean), 2020, and 2021. (**A**) IPD cases by age group in 2018–2019 (mean), 2020, and 2021. (**B**) IPD cases by quarter in 2018–2019 (mean), 2020, and 2021. (**C**) IPD cases caused by PCV13 serotypes by quarter in 2018–2019 (mean), 2020, and 2021. (**D**) IPD cases caused by non-PCV13 serotypes by quarter in 2018–2019 (mean), 2020, and 2021. Abbreviations: IPD, invasive pneumococcal disease. PCV13, 13-valent pneumococcal conjugate vaccine.

IPD incidence was 11.0 and 4.6 per 100,000 PY in 2018–2019 and 2021, respectively, representing a decrease of 58% (*p* < 0.001) overall, focused especially in the 0–4 age group (−56%; *p* = 0.002) (Table 1; Figure 1A). In the first quarters, reductions were 88% (*p* < 0.001) (Table 2). In 2021, compared to 2020, IPD incidence overall was broadly the same (4.6 and 3.8 per 100,000 PY, respectively), but decreased significantly in the first quarter, from 2.9 to 0.4 per 100,000 PY (−86%; *p* < 0.001) and increased in the second quarter, from 0.0 to 1.2 per 100,000 PY (*p* = 0.02) (Table 1 and Table 2; Figure 1B).

IPD incidence per 100,000 PY caused by PCV13 serotypes was 4.8 in 2018–2019 and 1.6 in 2021, reflecting a drop of 68% (*p* = 0.003). This decrease was higher than that for non-PCV13 serotypes, i.e., 5.6 and 2.9 per 100,000 PY in 2018–2019 and 2021, respectively, reflecting a drop of 48% (*p* = 0.04), with serotype 3 showing a decrease of 76% (*p* = 0.005). In 2021, compared to 2018–2019, the decrease in IPD incidence for PCV13 serotypes was only statistically significant for the 0–4 age group (−70%; *p* = 0.01), and, in this same age group, the greatest reduction was for serotype 3 (−82%; *p* = 0.01) (Table 1). As for 2021, compared to 2020, there were no significant changes in IPD incidence for the PCV13 and non-PCV13 serotypes, either overall or by age group. However, in the first quarter of 2021, incidence decreased for PCV13 serotypes, from 1.7 to 0.2 per 100,000 PY (−89%; *p* = 0.01), and also for serotype 3, from 1.5 to 0 per 100,000 PY (*p* = 0.01) (Table 1 and Table 2; Figure 1C,D). In contrast, in the second and fourth quarters of 2021 compared to 2020, IPD incidence increased overall, from 0.0 to 1.2 per 100,000 PY (*p* = 0.02) in the second quarter, and from 0.6 to 2.1 per 100,000 PY (+272%; *p* = 0.03) in the fourth quarter.

**Figure 2:**
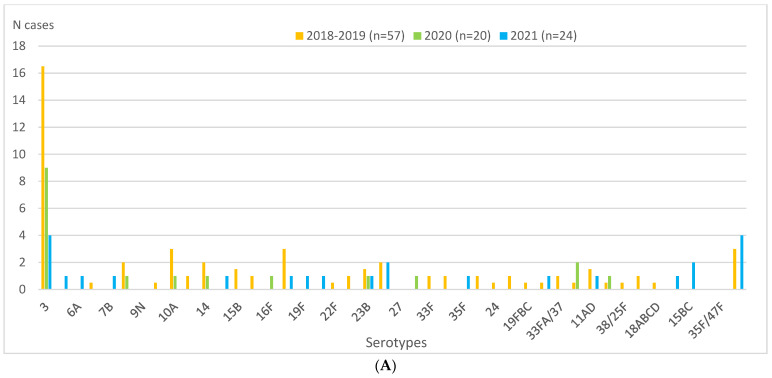

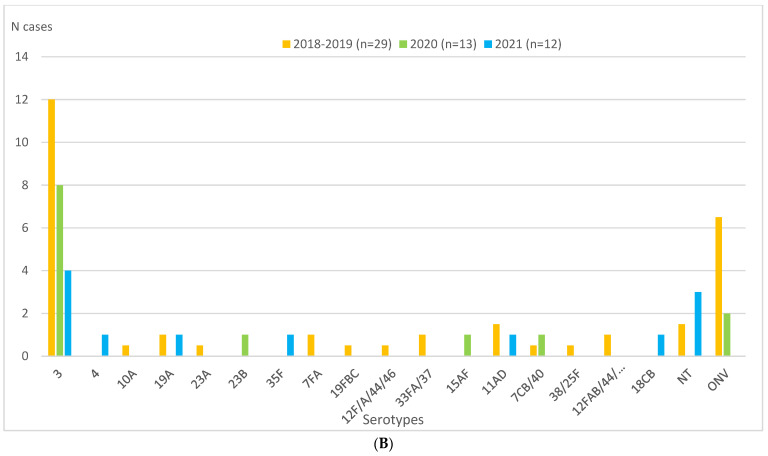
Children aged 0–17 years in Catalonia, Spain. Serotype distributions of IPD cases overall and diagnosed by PCR in 2018–2019 (mean), 2020, and 2021. (**A**) Serotype distribution of IPD cases in 2018–2019 (mean), 2020, and 2021. (**B**) Serotype distribution of IPD cases diagnosed exclusively by PCR in 2018–2019 (mean), 2020, and 2021. Abbreviations: IPD, invasive pneumococcal disease. NT, not typed. ONV, other non-vaccine serotypes. PCR, polymerase chain reaction.

During the entire study period, half or more of IPD cases were diagnosed exclusively by PCR: 50.0% (29/57) in the 2018–2019 pre-pandemic period, 65% (13/20) in 2020, and 50.0% (12/24) in 2021 (Table 3). The most frequent serotype throughout the period was serotype 3, representing 29.8%, 45%, and 16.7% of cases in 2018–2019, 2020, and 2021, respectively (Table 1 and Figure 2A). Serotype 3 was exclusively diagnosed by PCR in 70.6% (12/17), 88.9% (8/9), and 100% (4/4) of cases in 2018–2019, 2020, and 2021, respectively (Figure 2A,B).

The only serotype besides serotype 3 that was detected during the entire study period was serotype 23B (five cases, representing 2.6% of the total in 2018–2019, 5% in 2020 and 4.2% in 2021). Diagnosis of this serotype was exclusively by PCR in 2020 (one case), exclusively by culture in 2018–2019 (three cases), and by both techniques in 2021 (one case). 

## 4. Discussion

**Figure 3:**
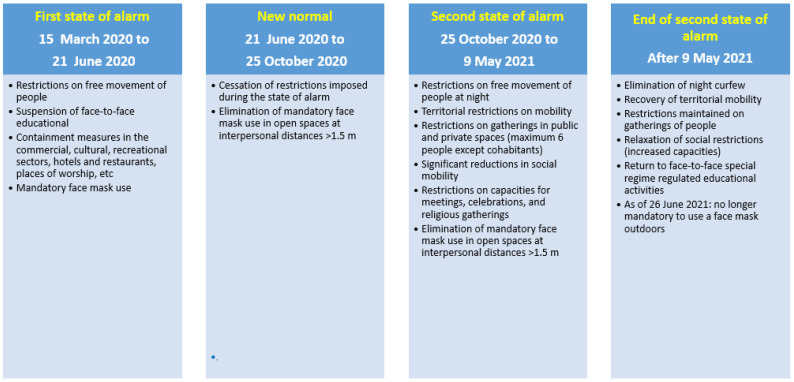
Restrictions and other public health measures imposed due to the COVID pandemic during the study period. First state of alarm [9], New Normal [10], Second state of alarm [11,12], End of de second state of alarm [13].

Government crisis management during the COVID-19 pandemic evolved depending on waves, with restrictive measures implemented in Spain as appropriate to reduce social contact and the spread of the virus (Figure 3). The Spanish government, in declaring the first state of alarm [9], imposed a strict lockdown from 14 March 2020 to 21 June 2020; by the third quarter of 2020, however, territorial mobility restrictions were relaxed in the so-called new normal period [10]. However, on 25 October 2020, a second state of alarm was declared that would last until 9 May 2021, with restrictions on territorial mobility, a night curfew, limitation on numbers attending social and religious gatherings, etc. [11,12]. As of 9 May 2021, a new resolution of the Government of Catalonia [13] came into force that extended opening hours and capacity numbers. Use of a mask in enclosed places continued to be generally mandatory, however, up to April 2022 [14], at which point this obligation ceased except for in healthcare centres, nursing homes, and on public transport, and responsible use continued to be recommended in relation to vulnerable persons.

Our results show that in 2021, compared to the 2018–2019 pre-pandemic period, there was a reduction in paediatric ER visits; this decrease was more pronounced in the first quarter, when the second state of alarm was in force. In 2021, compared to 2018–2019, there was no significant reduction in PCR test requests for IPD overall, although reductions were observed in the first and fourth quarters of 2021. Noteworthy was the fact that, in 2021, the reduction in PCR test requests was much lower than the reduction in IPD incidence in the first quarter; this would suggest that the reduced IPD incidence observed in the first quarter of 2021 compared to the pre-pandemic period was not because of fewer diagnoses due to the healthcare changes imposed by the pandemic, but because of the social mobility restrictions imposed until 9 May 2021 by the second state of alarm [11,12]. As for the remaining quarters, the reduction in IPD incidence in 2021 compared to the pre-pandemic period was not significant, probably due to the removal of social distancing restrictions with the ending of the second state of alarm in May 2021 [13], which, in turn, led to a recovery in IPD incidence.

Comparing 2021 with 2020, paediatric ER visits and PCR test requests for IPD increased overall, most especially in the second quarter once the second state of alarm ended (9 May 2021). However, a significant decrease was observed in the first quarters of 2021 compared to 2020, possibly due to the fact that lockdown was not imposed until 15 March 2020 and that certain restrictions associated with the second state of alarm were in force until 9 May 2021. The reductions in healthcare activity, along with the restrictions of the second state of alarm [11,12,13], would explain the significant decrease in IPD incidence observed in the first quarter of 2021 compared to the same quarter of 2020. The relaxation in social mobility restrictions with the ending of the second state of alarm [13] may explain the increased healthcare activity and increased IPD incidence observed in the second and fourth quarters of 2021 compared to 2020. 

Contrasting with our findings, some authors have reported a recovery in IPD incidence from the second quarter of 2021. In Germany, Perniciano et al. [6], in a study carried out from 1 January 2015 to 30 November 2021, found that IPD cases reached pre-pandemic levels in June 2021, although the increasing trend had already started in the spring of 2021. In Switzerland, Casanova et al. [5] studied IPD evolution from January 2017 to June 2021, confirming an increase from March 2021 and return to 2017–2019 levels by the end of June (coinciding with the easing of non-pharmacological anti-COVID-19 measures). In England, Bertran et al. [7], in a comparison of the second halves of 2021, 2020, and 2017–2019, reported a gradual increase in incidence in children aged <15 years from February 2021, with the increase exceeding pre-pandemic levels from July to December. 

In Catalonia, paediatricians began to recommend PCV13 in 2010 and, in 2016, included the 2 + 1 regimen for children aged <5 years and populations at risk in its systematic vaccination schedule. By 2020, 91% coverage was achieved with the complete regimen [15]. In 2018, 2019, and 2020 in Catalonia, PCV13 serotypes in children aged <5 years were 26.6%, 31.1%, and 25.7%, respectively [3]. In our study, for children aged <5 years, we found that PCV13 serotypes represented 40.9%, 53.3%, and 27.7% in 2018–2019, 2020, and 2021, respectively. The differences observed regarding the total number of cases notified in Catalonia may be explained by the fact that, in our study, culture and PCR tests were performed in 93% and 58% of cases, respectively, thus increasing the percentage of IPD cases diagnosed by negative culture (52.5%; 83/158) of all diagnosed cases); in Catalonia as a whole, diagnosis exclusively by PCR represented 4.7% for all age groups in 2019–2020 [3]. This difference is especially relevant for serotype 3, which, for children aged <5 years in our study, represented 27.3%, 53.3%, and 11.1% of diagnoses in 2018–2019, 2020, and 2021, respectively. Previous studies show that a large percentage of serotype 3 IPD cases are diagnosed exclusively by PCR [16,17]. 

In 2021, the reduction in IPD compared to the pre-pandemic period (2018–2019) was greater for PCV13 serotypes than for non-PCV13 serotypes, most especially for serotype 3. This was even more markedly the case for children aged <5 years, possibly partially attributable to both lower *S. pneumoniae* circulation (as a consequence of the non-pharmacological restrictions in place) and the impact of the PCV13 vaccine (included in the 2016 2 + 1 regimen for children aged <5 years). In 2021, compared to 2020, there were no significant variations overall or by age group, although there was a large decrease in the first quarter, especially in vaccine serotypes, and an increase in the second and fourth quarter for all serotypes. This is possibly explained by the fact that, in relation to the first quarters, restrictions had not yet been implemented in 2020, whereas in 2021, the second state of alarm was in force, and in relation to the second quarters, the situation was reversed, with a strict lockdown in 2020 but a relaxation of restrictions in 2021.

In Switzerland, in a study that covered all ages in 2019, Casanova et al. [5] observed that only 29.5% of the serotypes were PCV13, with serotype 3 being the most frequent (16.3%). The same authors reported a decrease in the proportion of PCV13 serotypes (24.8%) in the first half of 2021, with serotype 3 nonetheless continuing to be highly prevalent (15%); they also detected the emergence of the 23B serotype (prevalence of 3.2% in 2020 and 8% in 2021). Our higher percentage of IPD cases due to PCV13 serotypes than that reported by Casanova et al. [5] may be partly explained by the latter’s non-use of PCR as a diagnostic technique. Corroborating that same study, we found that serotype 3, although it decreased in 2021 compared to 2018–2019 (incidences of 0.8 and 3.3, respectively), continued to be by far the most frequent serotype (16.6% of the total) (Figure 2A,B). Regarding the 23B serotype, we observed that, although the number of cases was lower in 2020 and 2021 (one in each year) than in the 2018–2019 pre-pandemic period (two annual cases on average), the percentage increased each year (2.5% in 2018–2019, 5% in 2020, and 4.2% in 2021) (Figure 2A).

Perniciano et al. [6] observed that, in Germany, the proportions of serotypes covered by PCV13 remained constant in the overall population and in different age groups between 2015 and 2021. That study, like ours, reported serotype 3 to be the most common serotype, although in lower percentages (16–21% of all IPD cases in the pre-pandemic period, and 19% in 2020) than those reported by us, but slightly higher in 2021 (20%). The lower percentages of IPD cases due to PCV13 serotypes observed by Perniciano et al. [6] may also be explained by the non-use of PCR as a diagnostic technique. 

Other authors have highlighted the 23B serotype as both a cause [5] and carrier of IPD. Alfayate et al. [18], in the region of Murcia (Spain), compared pneumococcus carrier status in children aged 1–4 years 5 years after PCV13 vaccine introduction (summer 2014 and winter 2015); they reported that the carrier percentage was 19.8%, that only 14.4% were PCV13 serotypes, and that 23B was the most frequent serotype (13%). Skosana et al. [19], in a South African study of healthy children aged <5 years conducted between 2014 and 2016 (a decade after the introduction of conjugate vaccines), found that 43.7% were colonized by pneumococcus, most especially the younger cohorts. Non-PCV13 serotypes were reported in that study to be the most frequent serotypes (81.8%), especially 23B (13.2%) and 15B/C (11.6%). Patel et al. [20], in Botswana, analysed carrier status in children aged <24 months between 2012 and post-PCV13 vaccine introduction in 2017, finding that the proportion of PCV13 serotypes decreased from 70% in 2012–2013 to 25% in 2016–2017. Comparing the two periods, the same authors reported the most frequent serotypes to be 6A/6B (20%) in 2012–2013, and 23B (28.4%) in 2016–2017. Kielbik et al. [21], in Poland, conducted a carrier study in 2020 in children aged 1–6 years 3 years after introducing PCV10, finding pneumococcal colonization in 23.3% of children, reporting serotype 23B in 28% and 25% of the isolates in vaccinated and non-vaccinated children, respectively, and PCV13 serotypes in 17.1% of the isolates.

A limitation of our study is that, although the population is representative of the studied area, the sample size for some of the variables was small and so did not allow significant differences to be detected. 

As for its strengths, our study was carried out using the same methodology for the COVID-19 pre-pandemic and pandemic periods and the use of PCR as a diagnostic technique increased IPD diagnostic sensitivity. Finally, the comparison by quarters enabled us to both determine IPD seasonality [22,23,24] and highlight the role played by pandemic-related mobility restrictions.

## 5. Conclusions

In 2021, IPD incidence was observed to decrease in relation to 2018–2019, and this decrease was greater than the decrease in healthcare demand and in diagnostic test requests. IPD incidence decreased mainly in the first quarter of 2021, probably because, with the second state of alarm in force, social mobility continued to be restricted in 2021 compared to 2020. IPD was observed to decrease in the first quarter, when the second state of alarm was in force, and to increase from the second quarter, when the state of alarm ended. Pre-pandemic levels of IPD incidence were not reached in 2021.

The use of diagnostic techniques such as PCR, which is more sensitive than culture, results in more cases being detected (most especially serotype 3 cases) and also provides a better picture of IPD epidemiology. Surveillance of serotypes that cause IPD to circulate in the community is crucial to establish appropriate vaccination strategies.

## Figures and Tables

**Table 1:** Children aged 0–17 years in Catalonia, Spain. Healthcare activity and IPD incidence in 2018–2019 (mean), 2020, and 2021, for age groups 0–17 years, 0–4 years, and 5-17 years.

	Mean 2018–2019	2020	2021	2021 vs. 2018–2019	*p* Value	Variation	2021 vs. 2020	*p* Value	Variation
	N (IR)	N (IR)	N (IR)	IRR (CI 95%)		%	IRR (CI 95%)		%
0–17 years									
Emergency visits	227,148 (43,661.3)	148,637 (28,437.6)	178,243 (34,570.5)	0.79 (0.78–0.80)	<0.0001	−21	1.22 (1.21–1.22)	<0.0001	+22
PCR samples (HSJD, HIVH)	641 (123.2)	497 (95.1)	605 (117.3)	0.95 (0.85–1.06)	0.39	NS−	1.23 (1.10–1.39)	0.0005	+23
IPD cases	57 (11.0)	20 (3.8)	24 (4.6)	0.42 (0.26–0.68)	<0.0001	−58	1.22 (0.67–2.23)	0.52	NS+
PCV13 serotypes	25 (4.8)	10 (1.9)	8 (1.6)	0.32 (0.15–0.72)	0.003	−68	0.81 (0.32–2.05)	0.66	NS−
Serotype 3	17 (3.3)	9 (1.7)	4 (0.8)	0.24 (0.08–0.70)	0.005	−76	0.45 (0.14–1.46)	0.17	NS−
Non-PCV13 serotypes	29 (5.6)	10 (1.9)	15 (2.9)	0.52 (0.27–0.97)	0.04	−48	1.52 (0.68–3.51)	0.31	NS+
0–4 years									
Emergency visits	108,757 (93,016.7)	68,684 (60,617.9)	104,023 (96,896.3)	1.04 (1.03–1.05)	<0.0001	+4	1.60 (1.58–1.61)	<0.0001	+60
PCR samples (HSJD, HIVH)	459 (392.6)	342 (301.8)	449 (418.2)	1.07 (0.96–1.21)	0.34	NS+	1.38 (1.20–1.59)	<0.0001	+38
IPD cases	44 (37.6)	15 (13.2)	18 (16.8)	0.44 (0.25–0.76)	0.002	−56	1.27 (0.63–2.56)	0.50	NS+
PCV13 serotypes	18 (15.4)	8 (7.1)	5 (4.6)	0.30 (0.11–0.81)	0.01	−70	0.66 (0.22–2.02)	0.46	NS−
Serotype 3	12 (10.3)	8 (7.1)	2 (1.9)	0.18 (0.04–0.81)	0.01	−82	0.26 (0.06–1.24)	0.07	NS−
Non-PCV13 serotypes	25 (21.4)	7 (6.2)	12 (11.2)	0.52 (0.25–1.03)	0.06	NS−	1.80 (0.71–4.89)	0.22	NS+
5-17 years									
Emergency visits	118,391 (29,353.5)	79,953 (19,530.7)	74,220 (18,180.6)	0.62 (0.61–0.63)	<0.0001	−38	0.93 (0.92–0.94)	<0.0001	−7
PCR samples (HSJD, HIVH)	182 (45.1)	155 (37.9)	156 (38.2)	0.85 (0.68–1.05)	0.13	NS−	1.01 (0.81–1.26)	0.93	NS
IPD cases	13 (3.2)	5 (1.2)	6 (1.5)	0.46 (0.16–1.18)	0.11	NS−	1.20 (0.35–4.28)	0.77	NS+
PCV13 serotypes	7 (1.7)	2 (0.5)	3 (0.7)	0.42 (0.11–1.64)	0.20	NS−	1.50 (0.25–9.00)	0.65	NS+
Serotype 3	5 (1.2)	1 (0.2)	2 (0.5)	0.39 (0.08–2.04)	0.25	NS−	2.01 (0.18–22.12)	0.56	NS+
Non-PCV13 serotypes	4 (1.0)	3 (0.7)	3 (0.7)	0.74 (0.14–3.59)	0.72	NS−	1.00 (0.17–5.84)	0.99	NS

Abbreviations: CI, confidence interval. HSJD, Hospital Sant Joan de Déu. HIVH, Hospital Infantil Vall d’Hebron. IPD, invasive pneumococcal disease. IR, incidence rate per 100,000 person-years. IRR, incidence rate ratio. NS, non-significant. PCR, polymerase chain reaction. PCV13, 13-valent pneumococcal conjugate vaccine.

**Table 2:** Children aged 0–17 years in Catalonia, Spain. Healthcare activity and IPD incidence in 2018–2019, 2020, and 2021 by quarter.

Quarter	Mean 2018–2019	2020	2021	2021 vs. 2018–2019	*p* Value	Variation	2021 vs. 2020	*p* Value	Variation
	N (IR)	N (IR)	N (IR)	IRR (CI 95%)		%	IRR (CI 95%)		%
1 st quarter									
Emergency visits	61,590 (11,838.5)	54,430 (10,413.7)	37,324 (7239.0)	0.61 (0.60–0.62)	<0.0001	−39	0.70 (0.69–0.70)	<0.0001	−30
PCR samples (HSJD, HIVH)	185 (35.6)	182 (34.8)	98 (19.0)	0.53 (0.42–0.68)	<0.0001	−47	0.54 (0.43–0.70)	<0.0001	−46
IPD cases	17 (3.3)	15 (2.9)	2 (0.4)	0.12 (0.02–0.45)	0.0004	−88	0.14 (0.02–0.52)	0.0001	−86
PCV13 serotypes	7 (1.3)	9 (1.7)	1 (0.2)	0.14 (0.02–1.17)	0.06	NS−	0.11 (0.01–0.89)	0.01	−89
Serotype 3	5 (1.0)	8 (1.5)	0 (0)	-	0.03	(-Not calc)	-	0.01	(-Not calc)
Non-PCV13 serotypes	10 (1.9)	6 (1.1)	1 (0.2)	0.10 (0.01–0.60)	0.001	−90	0.17 (0.01–1.14)	0.07	NS-
2 nd quarter									
Emergency visits	55,519 (10,671.6)	23,025 (4405.2)	46,291 (8978.2)	0.84 (0.83–0.85)	<0.0001	−16	2.04 (2.01–2.07)	<0.0001	+104
PCR samples (HSJD, HIVH)	141 (27.1)	107 (20.5)	198 (38.4)	1.42 (1.14–1.76)	0.001	+42	1.88 (1.48–2.37)	<0.0001	+88
IPD cases	15 (2.9)	0 (0)	6 (1.2)	0.40 (0.14–1.02)	0.05	NS−	-	0.02	(+Not calc)
PCV13 serotypes	8 (1.5)	0 (0)	2 (0.4)	0.25 (0.05–1.19)	0.06	NS−	-	0.15	NS+
Serotype 3	6 (1.2)	0 (0)	1 (0.2)	0.17 (0.02–1.10)	0.06	NS−	-	0.31	NS+
Non-PCV13 serotypes	6 (1.2)	0 (0)	4 (0.8)	0.67 (0.17–2.46)	0.56	NS−	-	0.06	NS+
3 rd quarter									
Emergency visits	44,594 (8571.6)	34,933 (6683.5)	35,692 (6922.5)	0.81 (0.80–0.82)	<0.0001	−19	1.04 (1.02–1.05)	<0.0001	+4
PCR samples (HSJD, HIVH)	112 (21.5)	86 (16.5)	158 (30.6)	1.42 (1.12–1.81)	0.004	+42	1.86 (1.43–2.42)	<0.0001	+86
IPD cases	6 (1.2)	2 (0.4)	5 (1.0)	0.84 (0.24–2.88)	0.78	NS−	2.53 (0.50–18.86)	0.28	NS+
PCV13 serotypes	2 (0.4)	0 (0)	1 (0.2)	0.50 (0.05–5.56)	0.57	NS−	-	0.31	NS+
Serotype 3	1 (0.2)	0 (0)	0 (0)	-	0.32	NS−	-		NS
Non-PCV13 serotypes	3 (0.6)	2 (0.4)	4 (0.8)	1.34 (0.28–7.21)	0.72	NS+	2.03 (0.36–15.83)	0.44	NS+
4 th quarter									
Emergency visits	65,445 (12,579.5)	36,249 (6935.3)	58,933 (11,430.1)	0.91 (0.90–0.92)	<0.0001	−9	1.65 (1.963–1.67)	<0.0001	+65
PCR samples (HSJD, HIVH)	203 (39.0)	122 (23.3)	151 (29.3)	0.75 (0.61–0.92)	0.007	−25	1.25 (0.99–1.59)	0.06	NS+
IPD cases	19 (3.7)	3 (0.6)	11 (2.1)	0.58 (0.27–1.22)	0.16	NS−	3.72 (1.04–13.32)	0.03	+272
PCV13 serotypes	8 (1.5)	1 (0.2)	4 (0.8)	0.50 (0.15–1.67)	0.25	NS−	4.05 (0.45–36.28)	0.17	NS+
Serotype 3	5 (1.0)	1 (0.2)	3 (0.6)	0.60 (0.14–2.53)	0.49	NS−	3.04 (0.32–29.24)	0.31	NS+
Non-PCV13 serotypes	10 (1.9)	2 (0.4)	6 (1.2)	0.60 (0.20–1.67)	0.34	NS−	3.04 (0.64–21.89)	0.17	NS+

Abbreviations: CI, confidence interval. HSJD, Hospital Sant Joan de Déu. HIVH, Hospital Infantil Vall d’Hebron. IPD, invasive pneumococcal disease. IR, incidence rate per 100,000 person-years. IRR, incidence rate ratio. NS, non-significant. Not calc: Not calculable. PCR, polymerase chain reaction. PCV13, 13-valent pneumococcal conjugate vaccine.

**Table 3:** Children aged 0–17 years in Catalonia, Spain. IPD case distribution by diagnostic technique in 2018–2019 (mean), 2020, and 2021.

Diagnostic Technique	Mean 2018–2019	%	2020	%	2021	%	2018–2021	%
Culture only	16	28.9	2	10	4	16.7	38	24.1
PCR only	29	50.0	13	65	12	50.0	83	52.5
Culture + PCR	12	21.1	5	25	8	33.3	37	23.4
Total	57	100.0	20	100	24	100	158	100

Abbreviations: IPD, invasive pneumococcal disease. PCR, polymerase chain reaction.

## Data Availability

The data presented in this study are available on request from the corresponding author.

## References

[1] Kim Y.K., Choi Y.Y., Lee H., Song E.S., Ahn J.G., Park S.E., Lee T., Cho H.K., Lee J., Kim Y.J. (2022). Differential impact of nonpharmaceutical interventions on the epidemiology of invasive bacterial infections in children during the Coronavirus Disease 2019 Pandemic. Pediatr. Infect. Dis. J..

[2] Janapatla R.P., Chen C.L., Dudek A., Li H.C., Yang H.P., Su L.H., Chiu C.H. (2021). Serotype transmission dynamics and reduced incidence of invasive pneumococcal disease caused by different serotypes after implementation of non-pharmaceutical interventions during COVID-19 pandemic. Eur. Respir. J..

[3] Ciruela P., Broner S., Izquierdo C., Ayneto X., Coronas L., Muñoz Almagro C., Pallarès R., Ardanuy C., Martínez M., Cabezas C. (2022). Epidemiology of Invasive Pneumococcal Disease in Catalonia, Report 2019–2020. https://salutpublica.gencat.cat/ca/detalls/Article/malaltia-pneumococcica-invasiva-00001.

[4] Ciruela P., Soldevila N., García-Garcia J.J., González-Peris S., Díaz-Conradi A., Redin A., Viñado B., Izquierdo C., Muñoz-Almagro C., Domínguez A. (2022). Effect of COVID-19 pandemic on invasive pneumococcal disease in children, Catalonia, Spain. Emerg. Infect. Dis..

[5] Casanova C., Küffer M., Leib S.L., Hilty M. (2021). Re-emergence of invasive pneumococcal disease (IPD) and increase of serotype 23B after easing of COVID-19 measures, Switzerland, 2021. Emerg. Microbes Infect..

[6] Perniciaro S., van der Linden M., Weinberger D.M. (2022). Reemergence of invasive pneumococcal disease in Germany during the spring and summer of 2021. Clin. Infect. Dis..

[7] Bertran M., Amin-Chowdhury Z., Sheppard C.L., Eletu S., Zamarreño D.V., Ramsay M.E., Litt D., Fry N.K., Ladhani S.N. (2022). Increased incidence of invasive pneumococcal disease among children after COVID-19 pandemic, England. Emerg. Infect. Dis..

[8] Rocafort M., Henares D., Brotons P., Launes C., Fernandez de Sevilla M., Fumado V., Barrabeig I., Arias S., Redin A., Ponomarenko J. (2022). Impact of COVID-19 lockdown on the nasopharyngeal microbiota of children and adults self-confined at home. Viruses.

[9] Real Decreto 463/2020, de 14 de Marzo, por el que se Declara el Estado de Alarma para la Gestión de la Situación de Crisis Sanitaria Ocasionada por el COVID-19. BOE núm. 67, de 14 de Marzo de 2020, Páginas 25390 a 25400. https://www.boe.es/eli/es/rd/2020/03/14/463.

[10] Real Decreto-Ley 21/2020, de 9 de Junio, de Medidas Urgentes de Prevención, Contención y Coordinación para Hacer Frente a la Crisis Sanitaria Ocasionada por el COVID-19. BOE» núm. 163, de 10 de Junio de 2020, Páginas 38723 a 38752. https://www.boe.es/diario_boe/txt.php?id=BOE-A-2020-5895.

[11] Crisis Sanitaria Covid 19 Evolución de la Gestión de la Crisis en España. Gobierno de España. Real Decreto 926/2020, de 25 de Octubre, por el que se Declara el Estado de Alarma Para Contener la Propagación de Infecciones Causadas por el SARS-CoV-2. https://www.boe.es/buscar/act.php?id=BOE-A-2020-12898.

[12] Crisis Sanitaria Covid 19 Evolución de la Gestión de la Crisis en España. Real Decreto 956/2020, de 3 de Noviembre, por el que se Prorroga el Estado de Alarma Declarado por el Real Decreto 926/2020, de 25 de Octubre, por el que se Declara el Estado de Alarma para Contener la Propagación de Infecciones Causadas por el SARS-CoV-2. https://www.boe.es/buscar/act.php?id=BOE-A-2020-13494.

[13] Ondacero, 07.05.2021. https://www.ondacero.es/emisoras/catalunya/noticies/levanta-confinamiento-elimina-toque-queda-partir-9-mayo-estas-son-restricciones-vigor_20210507609527c86c3eec000114c862.html.

[14] La Moncloa Consejo de Ministros, 19.04.2022. https://www.lamoncloa.gob.es/consejodeministros/resumenes/Paginas/2022/190422-rp-cministros.aspx#:~:text=El%20Ejecutivo%20recomienda%20su%20uso%20responsable%20en%20la%20poblaci%C3%B3n%20vulnerable.&text=El%20Consejo%20de%20Ministros%20ha,partir%20del%2020%20de%20abril.

[15] Ministerio de Sanidad (2020). Gobierno de España. Cobertura de Vacunación. https://www.sanidad.gob.es/areas/promocionPrevencion/vacunaciones/calendario-y-coberturas/coberturas/docs/Todas_las_tablas2020.pdf.

[16] Izquierdo C., Ciruela P., Hernández S., García-García J.J., Esteva C., Moraga-Llop F., Díaz-Conradi A., Martínez-Osorio J., Solé-Ribalta A., de Sevilla M.F. (2020). Pneumococcal serotypes in children, clinical presentation and antimicrobial susceptibility in the PCV13 era. Epidemiol. Infect..

[17] Silva-Costa C., Brito M.J., Pinho M.D., Friães A., Aguiar S.I., Ramirez M., Melo-Cristino J., Portuguese Group for the Study of Streptococcal Infections, Portuguese Study Group of Invasive Pneumococcal Disease of the Pediatric Infectious Disease Society (2018). Pediatric complicated pneumonia caused by *Streptococcus pneumoniae* serotype 3 in 13-valent pneumococcal conjugate vaccinees, Portugal, 2010–2015. Emerg Infect Dis..

[18] Alfayate Miguélez S., Yague Guirao G., Menasalvas Ruíz A.I., Sanchez-Solís M., Domenech Lucas M., González Camacho F., Ortíz Romero M.M., Espejo García P., Guerrero Gómez C., Iofrío de Arce A. (2020). Impact of pneumococcal vaccination in the nasopharyngeal carriage of *Streptococcus pneumoniae* in healthy children of the Murcia Region in Spain. Vaccines.

[19] Skosana Z., Von Gottberg A., Olorunju S., Mohale T., Du Plessis M., Adams T., Mbelle N. (2021). Non-vaccine serotype pneumococcal carriage in healthy infants in South Africa following introduction of the 13-valent pneumococcal conjugate vaccine. S. Afr. Med. J..

[20] Patel S.M., Shaik-Dasthagirisaheb Y.B., Congdon M., Young R.R., Patel M.Z., Mazhani T., Boiditswe S., Leburu T., Lechiile K., Arscott-Mills T. (2022). Evolution of pneumococcal serotype epidemiology in Botswana following introduction of 13-valent pneumococcal conjugate vaccine. PLoS ONE.

[21] Kielbik Kielbik K., Pietras A., Jablonska J., Bakiera A., Borek A., Niedzielska G., Grzegorczyk M., Grywalska E., Korona-Glowniak I. (2022). Impact of pneumococcal vaccination on nasopharyngeal carriage of *Streptococcus pneumoniae* and microbiota profiles in preschool children in South East Poland. Vaccines.

[22] Janoff E.N., Musher D.M., Bennet E.J., Dolin R., Blaser M.I. (2020). Streptococcus pneumoniae. Principles and Practice of Infectious Diseases.

[23] Heyman D.L. (2022). Control of Communicable Diseases Manual.

[24] Kimberlin D.W., Barnett E.D., Lynfield R., Sawyer M.H., American Academy of Pediatrics (2021). Streptococcus pneumoniae (Pneumococcal) infections. Red Bood 2021 Report of the Committee on Infectious Diseases.

[25] (2015). Decret 203/2015, de 15 de Setembre, pel Qual es Crea la Xarxa de Vigilància Epidemiològica i es Regulen els Sistemes de Notificació de Malalties de Declaració Obligatòria i els Brots Epidèmics. DOGC.

